# Exploring the Correlates of Hematological Parameters With Early Diabetic Nephropathy in Type 2 Diabetes Mellitus

**DOI:** 10.7759/cureus.39778

**Published:** 2023-05-31

**Authors:** Susmitha Chollangi, Nikunj K Rout, Sudhir K Satpathy, Bandita Panda, Shubhransu Patro

**Affiliations:** 1 Department of General Medicine, Kalinga Institute of Medical Sciences, Kalinga Institute of Industrial Technology Deemed to be University, Bhubaneswar, IND; 2 Department of Nephrology, Kalinga Institute of Medical Sciences, Kalinga Institute of Industrial Technology Deemed to be University, Bhubaneswar, IND; 3 Department of Research and Development, Kalinga Institute of Medical Sciences, Kalinga Institute of Industrial Technology Deemed to be University, Bhubaneswar, IND

**Keywords:** red cell distribution width, type 2 diabetes, type 2 diabetes mellitus, microalbuminuria, red blood cell distribution (rdw), neutrophil to lymphocyte ratio (nlr), diabetic nephropathy

## Abstract

Background: Type 2 diabetes mellitus (DM) with nephropathy is a common complication in poorly controlled diabetes. Uncontrolled DM leads to intraglomerular vascular changes that cause physical injury to capillary walls, causing a profibrotic response in kidneys. The present study aimed to determine the association of hematological markers with microalbuminuria in early diabetic nephropathy.

Methods: A single-center, cross-sectional study was conducted over the period of two years at the Department of Medicine of Pradyumna Bal Memorial Hospital, Kalinga Institute of Medical Sciences. A total of 90 patients diagnosed with type 2 DM were classified into two groups (group A and group B) according to microalbuminuria; there were 45 patients in each group. Levels of hematological markers, i.e., neutrophil-to-lymphocyte ratio (NLR) and red cell distribution width (RDW), between the study groups were examined and compared.

Results: A significant difference in NLR was found between groups A and B (p = 0.001). A statistically significant difference in RDW was found between the groups (p = 0.015). Receiver operating characteristic curve analysis of inflammatory markers and microalbuminuria prediction showed an area under the curve of 0.814 for NLR and 0.656 for RDW.

Conclusion: Hematological parameters like NLR and RDW are elevated in early diabetic nephropathy patients. NLR is found to be a better marker than RDW in predicting early nephropathy.

## Introduction

Diabetes mellitus (DM) with persistently high blood sugar levels leads to microvascular (nephropathy, retinopathy, and neuropathy) and macrovascular (coronary artery disease, peripheral artery disease, and cerebrovascular accidents) complications [1]. The global prevalence of diabetes raised to 9.8%. In India, it is estimated that the number of cases of diabetes will be raised to 69.9 million by 2025 [2]. Early detection of microvascular problems would alert us to an elevated risk of cerebrovascular and cardiovascular consequences [3]. Many inflammatory cytokines, including interleukin-1 (IL-1), interleukin-8 (IL-8), interleukin-6 (IL-6), and tumor necrosis factor (TNF), play an important role in the development and progression of diabetic nephropathy [4]. The analysis of these cytokines is expensive and due to technical limitations in measuring these inflammatory indicators, they are not routinely employed in clinical practice. Whereas hematological parameters such as neutrophil-to-lymphocyte ratio (NLR) and red cell distribution width (RDW) are cheaper, cost-effective, and easy to measure as routine blood tests [5,6]. White blood cell count along with differential cell count are being studied as an inflammatory marker for many days. Neutrophil-mediated innate immune response (carried by neutrophils) and adaptive immune response (carried by lymphocytes) are both reflected in the NLR index.

Hyperglycemia has a range of effects on red blood cells (RBCs), including hemoglobin glycation, decreased deformability, and decreased longevity. RDW is used in clinical practice to discriminate amongst causes of anemia, and it is included in the majority of regular hematological investigations [7]. High RDW values were found to be related to an elevated risk of cardiovascular disease and nephropathy [8]. Higher RDW levels are associated with decreased estimated glomerular filtration rate (eGFR), greater proteinuria, lower levels of albumin and hemoglobin, and more severe glomerular damage [9]. Studies on the association of NLR and RDW in uncontrolled diabetic patients with microalbuminuria are limited. Thus, the present study aimed to find out the role of hematological parameters, i.e., NLR and RDW, in uncontrolled diabetes with or without microalbuminuria.

## Materials and methods

An observational study was conducted in the Department of Medicine, Kalinga Institute of Medical Sciences, during the period from December 2020 to July 2022 after obtaining approval from the Research and Institutional Ethics Committee, Kalinga Institute of Medical Sciences (KIIT/KIMS/IEC/472/2020). The sample size was calculated as 90 with 45 patients per group by using power calculation, with a power of the test of 85% and a 5% level of significance. All diabetic patients having hemoglobin A1c (HbA1C) of more than 7% with an age of more than 18 years were included in the study. Patients with acute and chronic infections, chronic inflammatory disease, immune disorders, cancer, hypertension, patients on anti-inflammatory drugs, angiotensin-converting enzyme inhibitors, angiotensin II receptor blockers, steroids, and patients diagnosed with kidney diseases were excluded from the study. Written consent was taken from each patient and a detailed history was taken followed by a thorough clinical and laboratory examination, including fasting blood sugar, post-prandial blood sugar, glycated HbA1C, complete blood count, NLR and RDW levels, urine routine, microscopy tests, and spot urine albumin to creatinine ratio (UACR).

Uncontrolled diabetic patients (HbA1C > 7%) were categorized into two groups based on microalbuminuria. Group A included 45 patients without microalbuminuria and group B had 45 patients with microalbuminuria.

Statistical analysis

Statistical analysis was conducted by using SPSS software version 25 (IBM Corp., Armonk, NY). Mean and standard deviation were used for continuous data and percentage data were presented for all the categorical data. The correlation between the parameters was expressed by their correlation coefficient and the cut-off value, and the specificity and sensitivity of the selected markers are expressed based on the receiver operating characteristics (ROC) curve.

## Results

In the total study population, the mean age was 62 years and as per gender distribution, 60% were male and 40% were female (Table 1). Biochemical parameters such as HbA1C, serum creatinine, UACR, high-density lipoprotein cholesterol (HDL-C), and low-density lipoprotein cholesterol (LDL-C) were recorded and their levels are presented in Table 1. The result shows that in uncontrolled diabetes with or without microalbuminuria, the mean HbA1C ranged between 8.25 ± 0.92 and 8.84 ± 1.85. In group B, the mean serum creatinine was 1.10 ± 0.26 mg/dl, whereas, in group A, it was 0.87 ± 0.27 mg/dl. UACR was measured as 201.77 ± 55.71 mg/g in group B, whereas in group A, the UACR level was 12.23 ± 3.62 mg/g. In group B, the mean triglycerides to high-density lipoprotein (TG/HDL) ratio was 4.68 ± 0.91 mmol/mol (82 mg/dl), and the mean LDL was 88.46 mg/dl. The level of high-density lipoprotein (HDL) in group A was 4.31 ± 1.05 mmol/l (77 mg/dl) and the mean low-density lipoprotein (LDL) was 88.36 mg/dl, respectively.

**Table 1:** Clinico-demographic features of uncontrolled diabetes with and without microalbuminuria DM: diabetes mellitus; HbA1c: hemoglobin A1C; Urine ACR: urine albumin creatinine ratio; TG/HDL: triglycerides/high-density lipoprotein; LDL-C: low-density lipoprotein cholesterol.

Clinico-demographic features	Group		P-value (p = 0.05) of the t-test for the significance level
	Uncontrolled DM without microalbuminuria (N = 45), Group A	Uncontrolled DM with microalbuminuria (N = 45), Group B	
Age (years) (mean ± SD)	62.40 ± 9.9	62.09 ± 9.6	0.881 (NS)
Gender, female, N (%)	19 (42.2)	18 (40)	0.830 (NS)
Male, N (%)	26 (57.8)	27 (60)	
HbA1c (%)	8.25 ± 0.92	8.84 ± 1.85	0.059 (NS)
Serum creatinine (mg/dl) (mean ± SD)	0.87 ± 0.27	1.10 ± 0.26	0.000 (S)
Urine ACR (mg/g) (mean ± SD)	12.23 ± 3.62	201.77 ± 55.71	0.000 (S)
TG/HDL ratio, median (interquartile range)	4.57 (3.58-4.88)	4.65 (4.00-5.48)	0.224 (NS)
LDL-C (mg/dl), median (interquartile range)	89.43 (81.36-96.30)	89.00 (79.20-95.90)	0.818 (NS)

Hematological parameters in uncontrolled diabetes with and without microalbuminuria

In hematological parameters, NLR and RDW were recorded and the data are presented in Table 2. The result shows that no clinical significance was observed in the mean value of RDW within the group although statistically representing significance with a p-value of 0.015. The mean value of NLR was significantly higher in diabetic patients with microalbuminuria (2.61 ± 0.44) as compared to those without microalbuminuria (2.06 ± 0.40) with a p-value of 0.000.

**Table 2:** Comparison of the neutrophil-to-lymphocyte ratio (NLR) and red cell distribution width (RDW) in both groups A and B for microalbuminuria differentiation DM: diabetes mellitus.

Hematological parameters with a normal range	Group		P-value of the t-test (p = 0.05 for significance level)
	Uncontrolled DM without microalbuminuria (N = 45), Group A	Uncontrolled DM with microalbuminuria (N = 45), Group B	
	Mean ± SD	Mean ± SD	
RDW (11.6-14.0)	14.30 ± 0.85	14.75 ± 0.91	0.015
NLR (1-2)	2.06 ± 0.40	2.61 ± 0.44	0.000

Correlation of NLR and RDW within the groups

The correlation of NLR and RDW within the groups is furnished in Table 3. Correlation between NLR and RDW within the groups showed a weak positive correlation, which indicates that an increase in NLR results in an increase in the RDW value or vice versa. In group A without microalbuminuria, NLR and RDW showed a correlation of 0.052 (p = 0.734), which is not significant. Similarly, in group B with microalbuminuria, NLR and RDW showed a non-significant correlation of -0.188 (p = 0.216).

**Table 3:** Correlation of NLR and RDW within the groups DM: diabetes mellitus; NLR: neutrophil-to-lymphocyte ratio; RDW: red cell distribution width.

Group	Correlation between NLR & RDW	
Uncontrolled DM without microalbuminuria (Group A)	Pearson correlation (r)	0.052
	P-value	0.734
	N	45
Uncontrolled DM with microalbuminuria (Group B)	Pearson correlation (r)	-0.188
	P-value	0.216
	N	45

Correlation of RDW and NLR with variables (HbA1C, serum creatinine, UACR, TG/HDL, and LDL-C) within groups

The correlation of RDW with HbA1C, serum creatinine, UACR, TG/HDL, and LDL-C within groups was recorded. The correlation of RDW and serum creatinine showed a significantly weak negative correlation in both groups A and B (Table 4). This indicated that an increase in RDW values also results in a decrease in a weak manner in serum creatinine and UACR value or vice versa in group A. But in group B, the relationship between RDW and UACR is not significant. RDW with TG/HDL and LDL-C did not have any significant correlation within any of the groups.

**Table 4:** Correlation of RDW and NLR with hemoglobin A1C (HbA1C), serum creatinine (S. Creat), urine albumin creatinine ratio (ACR), triglycerides/high-density lipoprotein (TG/HDL) ratio, and low-density lipoprotein cholesterol (LDL-C) within groups ** Correlation is significant at the 0.01 level. * Correlation is significant at the 0.05 level. DM: diabetes mellitus; NLR: neutrophil-to-lymphocyte ratio; RDW: red cell distribution width.

Variables	Uncontrolled DM without microalbuminuria (Group A)				Uncontrolled DM with microalbuminuria (Group B)			
	RDW		NLR		RDW		NLR	
	r-value	p-value	r-value	p-value	r-value	p-value	r-value	p-value
HbA1C	-0.285	0.057	0.414**	0.005	-0.064	0.674	0.662**	0.000
S. Creat	-0.377*	0.011	0.064	0.676	-0.363*	0.014	0.575**	0.000
Urine ACR	-0.379*	0.010	0.185	0.223	-0.226	0.136	0.293	0.051
TG/HDL	0.018	0.905	0.237	0.118	-0.245	0.105	-0.026	0.866
LDL-C	0.03	0.847	0.054	0.726	0.083	0.586	0.045	0.767

The correlation of NLR with HbA1C, serum creatinine, UACR, TG/HDL, and LDL-C within groups was recorded. The correlation of NLR and HbA1C depicted a significantly moderate positive correlation of 0.414 (p = 0.005) in group A. In group B, the correlation of NLR and HbA1C depicted a significantly strong positive correlation with an r-value of 0.662 (p = 0.000). The correlation of NLR and serum creatinine and UACR depicted a significantly moderate positive correlation. But in group A, the correlation between NLR and serum creatinine is not significant (p = 0.676). NLR with TG/HDL and LDL-C did not have any significant correlation within any of the groups.

ROC curve analysis of RDW and NLR as a predictive marker for microalbuminuria in type 2 diabetic patients

**Figure 1:**
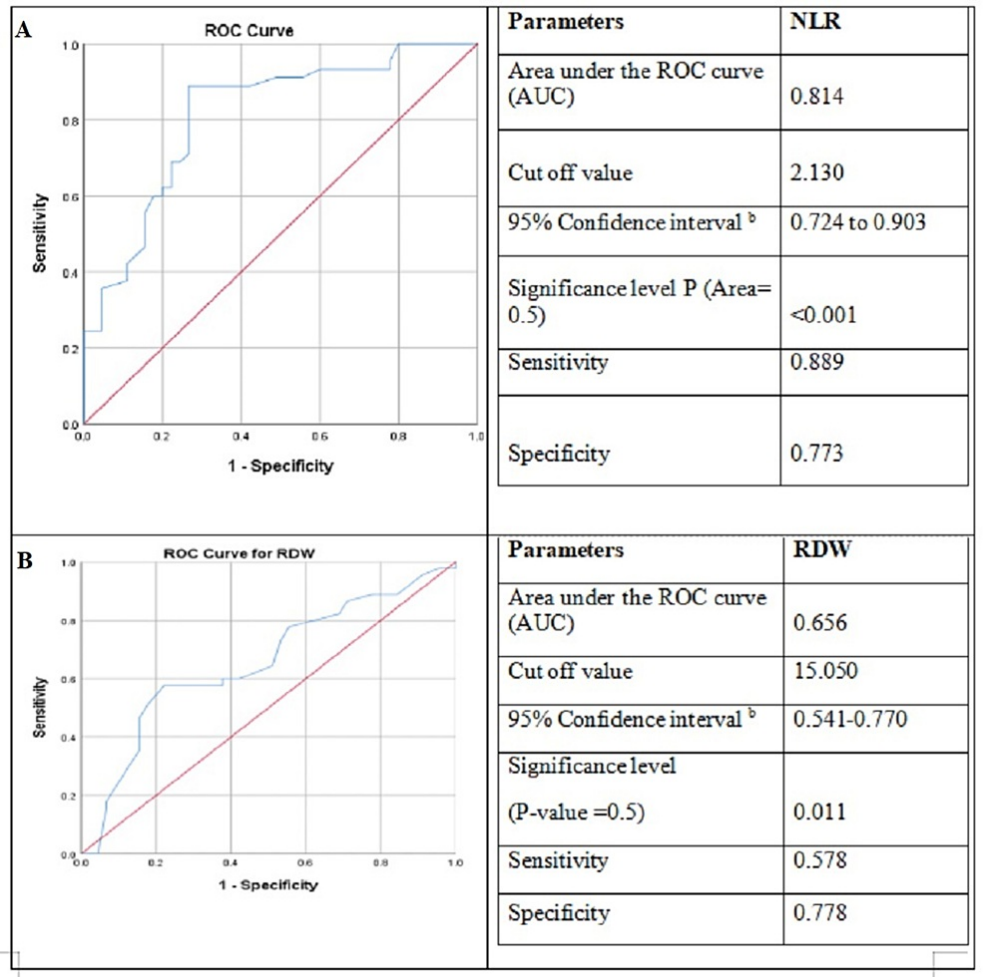
Sensitivity and specificity of NLR & RDW as predictive markers for microalbuminuria in type 2 diabetes patients A. Receiver operating characteristics (ROC) curve of neutrophil-to-lymphocyte ratio (NLR). B. ROC curve of red cell distribution width (RDW).

ROC analysis of RDW and NLR in groups A and B with 45 samples in each group was conducted for diabetic nephropathy prediction in type 2 diabetic patients in comparison to microalbuminuria level. The data revealed that the area under the curve (AUC) of NLR was 0.859 and the cut-off value for NLR was >2.18 (p < 0.001) with a sensitivity of 86.67 (95% CI ranged from 73.2 to 94.9) and the specificity was 77.78 (95% CI = 67.8-85.9) (Figure 1). The RDW has a cut-off value of >15 and an AUC was reordered to be 0.688 (p = 0.0003). The sensitivity was 57.78 with a 95% CI of 42.2-72.3. The specificity was 85.56 with a 95% CI of 76.6-92.1.

The sensitivity and specificity of NLR are high as compared to RDW, thus NLR may be considered as a good marker of microalbuminuria in type 2 diabetic patients.

## Discussion

The main pathology of uncontrolled type 2 diabetes is resistance to insulin in body tissues and/or defective insulin excretion from the pancreas. Because of persistent hyperglycemia and metabolic dysfunction, diabetes causes end-organ damage. The majority of the disease's morbidity and death is brought on by these consequences, which also lower patients' quality of life and place a heavy financial burden on them, their families, and the healthcare system.

A typical microangiopathic consequence in diabetic patients is diabetic nephropathy, which is the most typical cause of end-stage renal disease. Through immunologic inflammatory pathways, chronic inflammation plays a significant influence in the development and progression of type 2 diabetes. NLR and RDW, which are frequently obtained from standard hematological examination, may offer useful information for the early identification and assessment of diabetes-related complications like diabetic nephropathy, allowing for earlier management.

Keeping all these in view, this study was conducted to detect any correlation of blood parameters to identify early diabetic nephropathy in patients with DM. There was no significant difference between the groups as they were matched according to age. There was a slight male preponderance in the study population. This gender difference may be due to the random sampling method.

Patients were divided into groups based on their UACR values, and as expected, they differed in these parameters. In both groups, the mean HbA1c was around 8%. Creatinine was significantly higher in group B compared to group A with a mean creatinine being 0.87 ± 0.27 mg/dl in group A and 1.1 ± 0.26 mg/dl in group B (p < 0.001). This is similar to the studies conducted by Assulyn et al., Jaaban et al., Kahraman et al., and Afonso et al., but contrary to studies conducted by Zhang et al. [10-14].

This study's main purpose was to evaluate the hematologic indices' ability to predict microalbuminuria in type 2 diabetes individuals.

Our study showed significantly elevated levels of NLR in the “microalbuminuria group” compared with the other group (p < 0.001), with the mean NLR being 2.06 ± 0.40 in group A and 2.61 ± 0.44 in group B. This is according to similar studies conducted in Israel by Assulyn et al. and in China by Huang et al., where diabetic patients were selected as study subjects and grouped based on microalbuminuria [15]. The findings in our study were also similar to a study conducted on diabetic patients in Syria by Jaaban et al. where NLR was used as a marker for diabetic nephropathy, including both microalbuminuria and macroalbuminuria [11].

The findings are also similar to a study conducted in Turkey by Alsayyad et al. where NLR had a correlation with microalbuminuria and NLR was increased in patients with microalbuminuria irrespective of diabetic status [12]. The present study shows the cutoff value as 2.1 with 88% sensitivity and 73% specificity. Our result concurred with other studies where the best cutoff value for the diagnosis of NLR in diabetic nephropathy was 2.2 with 84% sensitivity and 98% specificity and is considered the best predictor and prognostic marker [12]. There are many studies correlating RDW with diabetic nephropathy but very few studies are correlating it with early diabetic nephropathy or microalbuminuria. Therefore, in our study, we compared RDW with microalbuminuria. Our study also showed elevated RDW in patients with microalbuminuria compared to patients without microalbuminuria with the mean being 14.30 ± 0.85 in group A and 14.75 ± 0.91 in group B. This finding is according to the study conducted in China by Zhang et al., which included a larger study population with 202 diabetic patients without microalbuminuria compared to 118 diabetic subjects with microalbuminuria, and to a study conducted in Detroit by Afonso et al. where the study was conducted in a large population of around 41,000 subjects who were segregated into quartiles based on RDW and showed significant correlation with UACR [13,14].

Our study also involves observing the pattern of dyslipidemia in the study groups. The lipid profile was observed for atherogenic dyslipidemia but there was no significant elevation in uncontrolled DM groups.

The limitation of the study was less to demonstrate a causal link between hematological parameters and microalbuminuria. More investigation is required to determine whether elevated hematologic indices are the cause or effect of early diabetic nephropathy.

## Conclusions

The study was conducted in the context of finding the correlation of NLR and RDW as hematological parameters with the parameters of diabetic nephropathy as well as finding a better marker between the two indices, which correlates more with early nephropathy. NLR level is higher in diabetic nephropathy patients than in RDW. NLR shows a significantly positive correlation with UACR in uncontrolled diabetes with microalbuminuria. NLR can be considered to be a better marker with a cutoff value of 2.1 than RDW in predicting early nephropathy.
